# Long-term organoid culture of a small intestinal neuroendocrine tumor

**DOI:** 10.3389/fendo.2023.999792

**Published:** 2023-04-04

**Authors:** Sabrina D’Agosto, Elena Fiorini, Francesco Pezzini, Pietro Delfino, Michele Simbolo, Caterina Vicentini, Silvia Andreani, Paola Capelli, Borislav Rusev, Rita T. Lawlor, Claudio Bassi, Luca Landoni, Antonio Pea, Claudio Luchini, Aldo Scarpa, Vincenzo Corbo

**Affiliations:** ^1^ Department of Diagnostics and Public Health, University of Verona, Verona, Italy; ^2^ Centre for Applied Research on Cancer (ARC-Net) Research Centre, University of Verona, Verona, Italy; ^3^ Azienda Ospedaliera Integrata dell’Università di Verona, Verona, Italy; ^4^ Pancreas Institute, Department of Surgery, University and Hospital Trust of Verona, Verona, Italy

**Keywords:** neuroendocrine tumor, small intestinal tumor, 3D culture, preclinical model, organoids

## Abstract

Gastroenteropancreatic neuroendocrine tumors (GEP-NETs) are rare and highly heterogeneous neoplasms whose incidence has markedly increased over the last decades. A grading system based on the tumor cells’ proliferation index predicts high-risk for G3 NETs. However, low-to-intermediate grade (G1/G2) NETs have an unpredictable clinical course that varies from indolent to highly malignant. Cultures of human cancer cells enable to perform functional perturbation analyses that are instrumental to enhance our understanding of cancer biology. To date, no tractable and reliable long-term culture of G1/G2 NET has been reported to permit disease modeling and pharmacological screens. Here, we report of the first long-term culture of a G2 metastatic small intestinal NET that preserves the main genetic drivers of the tumor and retains expression patterns of the endocrine cell lineage. Replicating the tissue, this long-term culture showed a low proliferation index, and yet it could be propagated continuously without dramatic changes in the karyotype. The model was readily available for pharmacological screens using targeted agents and as expected, showed low tumorigenic capacity *in vivo*. Overall, this is the first long-term culture of NETs to faithfully recapitulate many aspects of the original neuroendocrine tumor.

## 1 Introduction

Gastroenteropancreatic neuroendocrine neoplasms (GEP-NENs) are a group of heterogeneous malignancies arising from the neuroendocrine cells that are located in the pancreas or in different parts of the gastrointestinal tract. While the most recent epidemiologic data show a three-fold increase in their incidence over the last 40 years (1–5), GEP-NENs are still considered rare diseases. Surgical resection remains the only potentially curative treatment for patients with localized GEP-NENs. However, in most cases the disease is diagnosed at an advanced stage, precluding a curative treatment (1, 6, 7).

Histological differentiation and proliferation rate distinguish GEP-NENs with different clinical and biological behaviors. Indeed, neuroendocrine neoplasms showing a well-differentiated histology and low proliferation rate are commonly referred to as neuroendocrine tumors (NETs). Conversely, tumors displaying poor differentiation and a significant fraction of proliferative cells are called carcinomas (NEC). While carcinomas are invariably aggressive diseases (8–10), NETs are usually slow-growing neoplasms (11) that the latest WHO classification (12) categorized into 3 subtypes based on the proliferative index (e.g., mitotic counts or percentage of Ki67 positive cells): NET G1 (Ki67 index <3%), NET G2 (3%-20%), and NET G3 (>20%). High-grade tumors are invariably aggressive diseases, while low-to-intermediate grade neoplasms have a clinical and biological behavior that is difficult to predict. Due to the low incidence rate, consortium-based approaches have been instrumental in unraveling the major molecular underpinnings of these diseases (13–15). Key molecular drivers have been identified for Pancreatic Neuroendocrine Tumors (PanNET) (14, 16). In contrast, the genetic mechanisms underlying initiation and progression of small intestinal NET (siNET), which represent the most frequent cancer type of the small bowel, are still poorly understood (17–19). Genome-wide analyses of PanNETs showed a divergent genomic landscape dominated by *MEN1*, *ATRX* and *DAXX* alteration in NETs and by *TP53* and *RB1* in NECs (14, 15). Conversely, no recurrent altered genes were identified in siNET except for CDKN1B, which is mutated only in up to 10% of the cases (13, 17). The lack of target alterations makes it difficult to devise molecular targeted therapy as alternative to surgery in the case of advanced stage diseases. In keeping with this, a deeper molecular characterization of this disease beyond genomics or the full-exploitation of whole-genomes through innovative analytical approaches is urgently needed to expand the treatment options for the patients. Furthermore, the translation of research findings into the clinical practice has also been hampered by the lack of faithful models of the human disease for functional genomics. The available mouse and human GEP-NET cell lines are poorly representative of the genetics of human well-differentiated tumors (20). In a recent study published by Kawasaki and colleagues, successful derivation of long-term organoid cultures was reported only for G3 tumors but not for G1 and G2 NET samples (21), thus highlighting the difficulties of the *ex vivo* propagation of cells with limited proliferation capabilities. Accordingly, previous experiences have also demonstrated the inability of growing G1/G2 NETs upon xenotransplantation (22). Here, we have used the organoid culture methodology (23, 24) to develop a three-dimensional (3D) organoid culture from a G2 GI-NET. The model faithfully recapitulates the major molecular features of the tissue (including the proliferation rate), could be propagated extensively, and was used to test the activity of targeted agents. In keeping with its grade and differently from carcinomas, the model failed to establish detectable growth when transplanted in immunodeficient mice. Overall, our analyses suggest that this long-term culture faithfully recapitulate several aspects of the *in vivo* disease.

## 2 Materials and methods

### 2.1 Human specimens

Tumoral tissues were obtained from patients undergoing surgical resection at the Unit of General and Pancreatic Surgery of Verona University. This study was approved by the ethics committee at University of Verona, Italy: approval number 1911 (Prot. n 61413, Prog 1911 on 19/09/2018) from the Integrated University Hospital Trust (AOUI) Ethics Committee (Comitato Etico Azienda Ospedaliera Universitaria Integrata). Patient’s written informed consent was obtained prior to acquisition of the specimen. Samples were confirmed to be tumor based on pathological assessment.

### 2.2 Mice

All experimental procedures involving animals were performed according to protocols approved by the Institutional Animal Care and Use Committee at University of Verona (Approval number: 655/2017-PR). Six-to-eight weeks old NOD SCID gamma (NOD.Cg-Prkdc^scid^;Il2rg^tm1Wjl^; NSG; Charles River Laboratories) mice were used for this study. Mice were monitored daily and were euthanized according to Institutional-approved criteria.

### 2.3 Patient-derived organoid generation and culture

Fresh tissue specimens used in this study were collected from pancreatic resections. The material used to attempt at generating cultures was either from pancreatic tissue or from regional lymph node metastases (**Supplementary Table 1**). To confirm the presence of neoplastic cells within the tissues used for culture initiation, the pathologists processed each specimen to obtain two specular pieces of tissue. A mirror image was then obtained by cryostat sectioning of one of the two pieces followed by histopathological examination. After successful pathological assessment, tissue specimens were placed on ice in Human Splitting Medium [AdDMEM/F12 medium (Gibco) supplemented with HEPES (10 mM, Gibco), Glutamax™ (1X, Gibco), and Primocin (1 mg/ml, InvivoGen)] until they were transferred to the laboratory. Tissue samples were used to establish organoids culture using previously published procedures (23). Briefly, tissues were minced and digested with Collagenase II (5 mg/ml, Gibco) and Dispase I (1.25 mg/ml, Gibco), in Human Complete Medium (HCM) (see below) at 37°C for a maximum of 2 hours. An additional digestion was performed with TrypLE (Gibco) for 15 minutes at 37°C. Digested material was embedded in the appropriate volume of Growth-factor reduced Matrigel^®^ (Corning), and 50 µl of the suspension was plated in each well of a 24-well suspension plate (Greiner). Once solidified, the individual domes were overlaid with HCM (Human Splitting Medium supplemented with N-acetyl-L-cysteine (1.25 mM, Sigma), Wnt3a-conditioned medium (50% v/v), R-spo1-conditioned medium (10% v/v), recombinant Noggin (100 ng/ml, Peprotech), Epidermal Growth Factor (EGF, 50 ng/ml, Gibco), Gastrin (10 nM, Sigma), Fibroblast Growth Factor 10 (FGF10, 100 ng/ml, Peprotech), Nicotinamide (10 mM, Sigma), and A83-01 (0.5 µM, Tocris)). In addition to the standard pancreas organoid medium, Matrigel-embedded cells were also overlaid with experimental medium supplemented with additional growth factor or pathway inhibitors described in **Table 1**. The medium was changed every 3-4 days. For passaging, confluent organoid cultures were resuspended in Cell recovery solution (1X, Corning) and incubated for 30-60 minutes at 4°C to digest Matrigel. Subsequently, organoids were washed in Human Splitting Medium and mechanical dissociated to small cell clusters by pipetting. The resulting pellet was then resuspended in the appropriate volume of Matrigel to obtain a splitting ratio of 1:2. Given their low proliferative index, established siNET organoid cultures could be passaged on average once every 10 days. Organoid model labelled with the prefix HCM-CSHL was acquired as part of the Human Cancer Model Initiative (HCMI) https://ocg.cancer.gov/programs/HCMI and will be available for access from ATCC.

### 2.4 Histology and immunostaining

Sections of formalin-fixed, paraffin-embedded tissues and organoids were used for histopathological analyses. For embedding of the cultures, organoids of two individual Matrigel domes (total volume 100 µL) were liberated from the matrix using the Cell Recovery Solution (1X, Corning) as described in 2.3 and then fixed with Formalin for 20 minutes at room temperature. After washing out the fixative with PBS, the organoids were incubated with 70% Ethanol for 10 minutes and then embedded in Histogel Processing Gel (FisherScientific) and mostly placed in the same layer of the gel. Histogel-embedded cultures were then processed according to routine histology processing workflow. Haematoxylin and Eosin (H&E) staining was performed using standard protocols on 4 µm paraffin sections. Immunohistochemistry was performed following established procedures (25) using the reported primary antibodies: CK8-18 (5D3, Leica); CDX2 (EP25, Leica); CHGA (DAK-A3, Dako); SYP (27G12, Novocastra); CD56 (56C04, Thermofisher); Ki67 (ab16667, Abcam); ATRX (HPA001906, Sigma-Aldrich); DAXX (HPA008736, Sigma-Aldrich); p53 (NCL-L-p53-DO7, Novocastra); RB1 (RB-358-L-13A10). Slides were scanned at 20X magnification and digitalized using the Aperio Scan-Scope XT Slide Scanner (Aperio Technologies). To evaluate the percentage of Ki67 positive cells in the tissue specimen, two independent pathologists (B.R. and C.L.) manually counted a minimum of 500 nuclei from the regions showing highest labeling. To assess the percentage of Ki67 positivity in the organoid culture, the total number of nuclei and those showing positive staining were manually counted in 10 different fields of observation (magnification, 20X) to ensure counting a minimum of 500 nuclei.

### 2.5 Organoids dissociation into single cells

Organoids were incubated at 37°C for 20 minutes in Dispase I solution (Human Splitting Medium supplemented with 2 mg/ml of Dispase I) in order to digest Matrigel. Following, organoids were digested with TrypLE for 10 minutes at 37°C, incubated again in Dispase I solution supplemented with DNAse I (10 µg/ml) for additional 10 minutes, and pipetted several times to ensure single-cell suspension.

### 2.6 Karyotyping

For Karyotyping, organoids were incubated with 0.1 µg/mL Colcemid (Gibco) in HCM (as described in Boj et al. (23)) for 16 hours in humidified atmosphere at 37°C and 5% CO_2_. The following day, organoid cultures were dissociated into single cells as previously described. Single cells were incubated with KCL 0.0075 M hypotonic solution for 15 minutes, and fixed with two incubation of 15 minutes with methanol:acetic acid (3:1). Then cell pellets were washed twice with methanol:acetic acid (2:1), and dropped on a microscope slide for visualization. Nuclei were mounted and stained with DAPI (Vector Laboratories). A minimum of 15 metaphases per sample were counted.

### 2.7 Xenotransplantation

For transplantation, organoids were first dissociated as previously described (paragraph 2.5), and the cells counted. Recipient mice were anesthetized with isoflurane and an incision was made in the left abdominal side at the level of the spleen. One million cells resuspended in a volume of 50 µl of 60% v/v solution of Matrigel in cold PBS (Gibco) were injected into the pancreatic tail of each mouse using insulin syringes (BD micro-fine 30G). The peritoneum was sutured with short-term absorbable suture (Vetsuture), and the skin was closed with wound clips (CellPoint Scientific Inc.). Mice were monitored weekly by palpation and sacrificed three months post-injection. Pancreas, spleen, lungs, and liver were collected for downstream analysis.

### 2.8 *In vitro* treatment

For *in vitro* treatment, organoids were first dissociated as previously described (paragraph 2.5), and cells counted. 5000 cells per well were plated in 100µl of 10% Matrigel in HCM. Organoids were allowed to reform for two days and then treated with Everolimus (RAD001, S1120, Selleckchem), Palbociclib (PD0332991, Selleckchem), or vehicle control (DMSO). After 72 hours of treatment, cell viability was assessed using CellTiter-Glo^®^ (Promega) following manufacturer’s instruction. For activated caspase-3/7 detection, organoids were plated in 10% Matrigel in HCM and treated as previously described. The CellEvent™ Caspase-3/7 Green Detection Reagent (ThermoFisher) was added 24 hours before immunofluorescence evaluation. The pictures were taken using the EVOS™ M7000 Imaging System (ThermoFisher).

### 2.9 Whole exome sequencing

DNA was extracted from the organoid culture, the frozen tissue, and the associated blood specimen with DNeasy Blood and Tissue kit (Qiagen). Whole exome sequencing was performed using the SureSelect 50Mb capture kit (Agilent) and the NextSeq 500 Illumina platform. Average base-coverage was: 50X for the model, 150X for the tissue, and 30X for germline DNA. After quality checking and trimming with fastqc, whole exome sequencing data have been aligned with BWA. Then, sequenced reads were sorted and indexed, and duplicates marked with sambamba. Subsequently, recalibration and mutation calls were performed with Mutect2. Mutations were annotated with snpEffect, while mutational signatures were extracted with MuSiCa. Copy number variations were determined using the software tool Sequenza (26).

### 2.10 RNA sequencing

RNA was extracted from the organoid culture and the frozen tissue with PureLink RNA Mini Kit (Thermo Fisher Scientific) and subjected to poly(A) RNAseq library construction with TrueSeq Stranded mRNA Library Prep kit (Illumina). The resulting libraries were then sequenced to a depth of 30M fragments and 150 base paired-end reads on an Illumina NextSeq 500 sequencer. Raw FastQ files were subjected to quality control and quality cleaning with fastp, then a standard STAR/RSEM pipeline was employed to align reads to GRCh38 genome and to quantify reads against its corresponding transcriptome. Since no biological replicates were available for this specific case, to identify differentially expressed genes between the tissue and the patient-derived organoid (PDO) we made use of a larger matrix of expression profiles of PDAC tissues and corresponding organoids (data not shown). We used *edgeR* pipeline (27) to calculate a biological coefficient of variation (*BCV*) that ideally would represent an approximate biological variation for the siNET case. Squaring the *BCV* returned a dispersion value of 0.579 that was employed in the *exactTest* function to obtain *p-*values and fold-changes for each gene. For the correlation analysis all the genes identified after quantification were used, and nonparametric correlation was calculated. For Gene Set Enrichment Analysis (GSEA) we produced a ranked list of all the genes after *exactTest* according to log2 of fold change (PDO/tissue) and input the list to the *fgsea* function from *fgsea* package version 1.20.0 (28) with default parameters. Pathways used were from Molecular Signature Database (MSigDB) and in particular Gene Ontology, Reactome, KEGG, WikiPathways, Biocarta and Hallmarks (29). Pathways were considered significant for FDR < 0.1. All bioinformatics and statistical analysis were performed in the *R* environment version 4.0.5. All the plots were done with the *ggplot2* graphics library (https://ggplot2.tidyverse.org).

## 3 Results

### 3.1 Establishment of NET organoids

**Figure 1:**
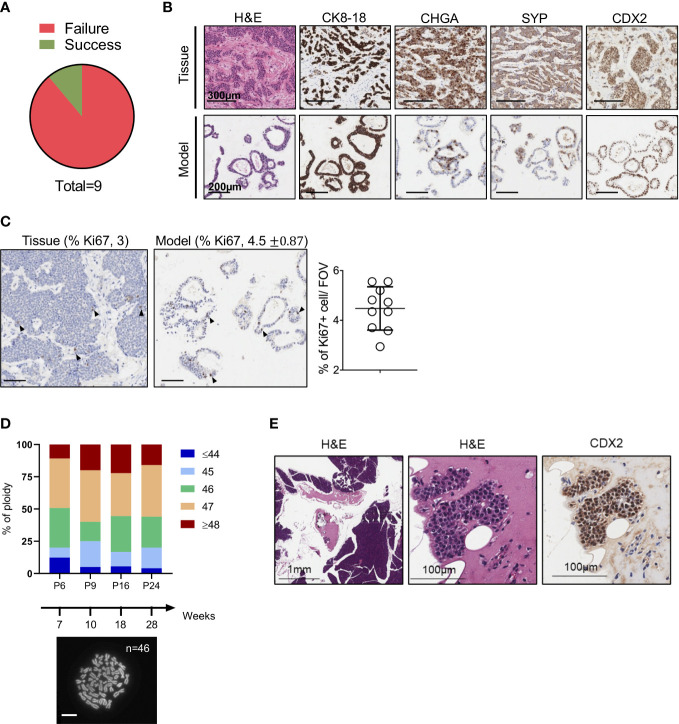
Establishment of NET Organoids. **(A)** Pie chart showing successful rate of organoids establishment from neuroendocrine tumor specimens. **(B)** Representative hematoxylin and eosin (H&E) staining and immunohistochemistry for cytokeratin 8-18 (CK8-18), chromogranin A (CHGA), synaptophysin (SYP), and caudal type homeobox 2 (CDX2) of the organoid culture and patient’s tissue (case HCM-CSHL-0608-17). Scale bars as indicated. **(C)** Representative immunohistochemistry for Ki67 of the organoid culture and the patient’s tissue. Scale bars, 200 µm. Quantification is provided at the top of the figure as percentage of Ki67 positive nuclei for both the tissue and the organoid culture. Standard Deviation is only shown for the organoid culture (see Methods). The scatter dot plot on the right displays the % of positive Ki67 nuclei in 10 fields of investigation (FOV, 20x magnification) for the organoid culture. **(D)** Ploidy analysis of the model at passage 6, 9, 16, and 24 over 28 weeks of continuous propagation. The proportion of metaphases with different number of chromosomes are represented by different colors as indicated in the legend. At the bottom, representative image of organoid metaphases used for the ploidy analysis. Scale bar, 10 μm. **(E)** Representative H&E staining and immunohistochemistry for CDX2 of the mouse pancreatic tissues collected 3 months after the injection of the organoid culture into the pancreas of immunocompromised mice. Scale bars as indicated.

We set up a NET patient-derived organoid (PDO) pipeline availing of resected tissues specimens from the University and Hospital Trust of Verona. Over 1.5-year, we collected a total of 9 tissue specimens that were suited for culture initiation and had a provisional diagnosis of well-differentiated neuroendocrine tumors of the pancreas (**Figure 1A**). For 8 out of the 9 cases, the diagnosis of well-differentiated PanNETs was confirmed based on histopathological assessment and immunophenotypic analysis (CDX2, CHGA, CK8-18, SYP, and Ki67) of formalin-fixed and paraffin-embedded (FFPE) tissues (**Supplementary Table 1**). Conversely, the clinical and pathological revision of the case HCM-CSHL-0608-C17 concluded that the tumor had originated from the small intestine (CDX2+, siNET) (**Figures 1B**, **S1A**), had metastasized to the pancreas, and displayed a proliferation index of 3% (G2 tumor) (**Figure 1C**, **Supplementary Table 1**). None of the other tumor tissues stained positive for the intestinal marker CDX2. Cultures were successfully initiated from all the 9 specimens using established procedures (see methods and (23)). Derived cell clusters were seeded in human pancreatic organoid media (23, 24) and in media supplemented with several growth factors (**Table 1**). Long-term propagation was exclusively observed for the HCM-CSHL-0608-C17, which was established from a loco-regional lymph node and could be propagated for 28 consecutive weeks in human complete media.

**Table 1:** Growth factors and pharmacological compounds used in organoid media.

Reagent	Source	Identifier	Final concentration	Function
Mouse recombinant EGF	Gibco	Cat # PMG8043	10 ng/ml	Growth factor
Human Fibroblast Growth Factor 10	PeproTech	Cat # 100-26	10 ng/ml	Growth factor
Murine Noggin	PeproTech	Cat # 250-38	100 ng/ml	BMP Pathway inhibitor
Dexamethasone	Sigma-Aldrich	Cat # D4902	3 nM	Cell culture supplement
Human Recombinant IGF-I	PeproTech	Cat # 100-11	100 ng/ml	Growth factor
DAPT	Sigma-Aldrich	Cat # D5942	10 μM	Notch Pathway inhibitor
Human Recombinant BMP7	PeproTech	Cat # 120-03P	25 ng/ml	Cytokine
A83-01	Tocris	Cat # 2939	500 nM	TGF-β Pathway inhibitor
N-acetyl-L-cysteine	Sigma-Aldrich	Cat # A9165	1.25 mM	Antioxidant
Gastrin I human	Sigma-Aldrich	Cat # 05-23-2301	10 nM	Hormone
Nicotinamide	Sigma-Aldrich	Cat # N0636	10 mM	Cell culture supplement
B27 supplement	Gibco	Cat # 17504001	1X	Supplement

Highlighted in grey, components not part of the standard pancreas organoid medium.

### 3.2 Clinicopathological features of HCM-CSHL-0608-C17

Morphological analysis of the organoid culture at different passages revealed acquisition of a cystic morphology (**Figures 1B**, **S1B**). In keeping with the immunophenotypical characterization of the tissue, the embedded organoid culture was assayed with the following markers: CDX2, CHGA, CK8-18, Ki67 and SYP. Prominent staining was observed for CDX2 and CK8-18, while a heterogeneous staining was observed in the organoid culture for the neuroendocrine markers CHGA and SYP (**Figures 1B**, **S1B**). A certain degree of heterogeneity for both the CHGA and the SYP staining could be observed in additional FFPE blocks from the same pancreatic tissue specimen (**Figure S1A**). As the culture was initiated from a loco-regional metastasis and no residual material was available for histopathological evaluation, we could not determine whether the heterogeneous expression of the two neuroendocrine markers is induced by the culture system, or it is rather an intrinsic feature of the starting material. To assess whether the established culture is truly representative of a G2 NET, we then performed immunohistochemical staining for the proliferative marker Ki67 and found that the fraction of positive cells was comparable between the culture (4.5%) and the tissue (3) (**Figure 1C**). Therefore, the model preserved the proliferation rate of the original tumor and could be classified as G2. Additionally, we found that the culture maintained a stable karyotype over the course of continuous passaging (**Figure 1D**). In line with previous findings, transplantation of siNETs into the pancreas of immunodeficient mice failed to generate growths (n = 6 mice) detectable at palpation. Three months following transplantation, pancreata were collected from transplanted mice and only few surviving CDX2+ cells could be seen at the injection site (**Figure 1E**, **S1C**). That is in stark contrast with the transplantation of a PDO from a neuroendocrine carcinoma (**Figure S1D**), which resulted in large tumor masses two months from injection into the mouse pancreata (**Figure S1E**). Overall, these results suggest low tumorigenic potential *in vivo* for the siNET PDO.

### 3.3 Genetic characterization of the siNET organoid culture

**Figure 2:**
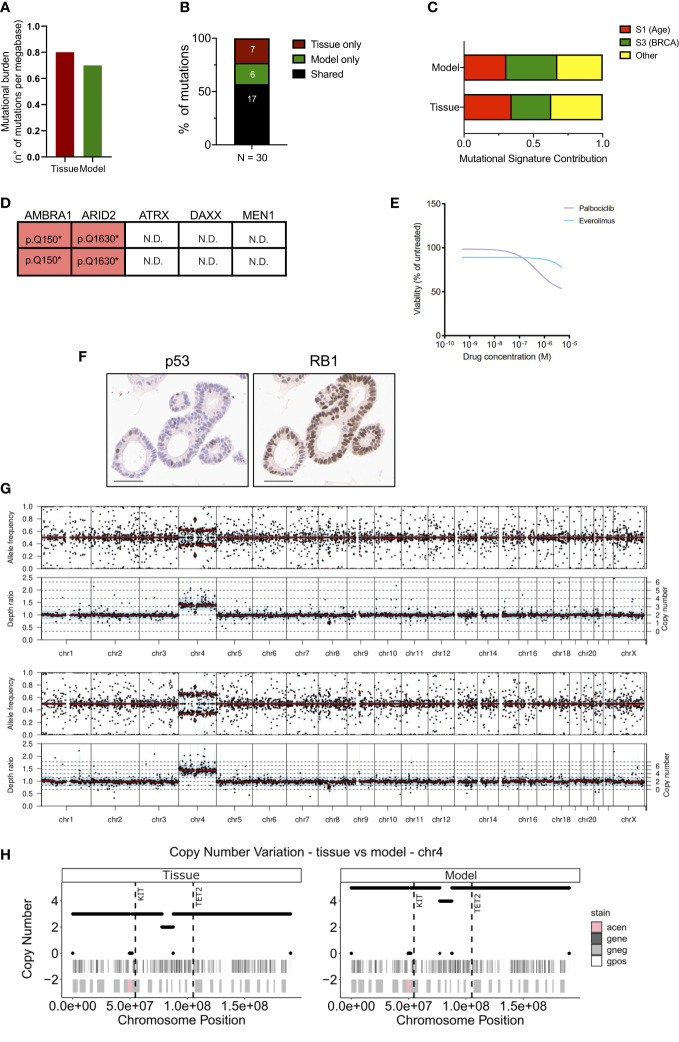
Genetic characterization of the siNET organoid culture. **(A)** Bar plot displaying the somatic mutations prevalence (number of non-synonymous mutations per Mb of DNA) in tissue and model. **(B)** Bar plot showing the proportion of shared (black) and private mutations identified in the model and tissue through whole-exome sequencing. Number of mutations is indicated. **(C)** Bar plot showing the concordance of the mutational signatures extracted from the analysis of patient’s tissue and derived organoid. **(D)** Mutational status of tumor suppressors and known neuroendocrine tumors drivers from whole-exome sequencing data. N.D., not detected. **(E)** Dose response curves of the PDO treated with Palbociclib or Everolimus. **(F)** Representative immunohistochemistry for p53 and RB1 in the organoid culture. Scale bar, 50μm. **(G)** Visualization of copy number variations (CNV) estimated by the software Sequenza for the tissue (top panel) and the model (bottom panel). Allele frequency and depth ratio raw profiles are reported according to genomic position. The mutant allele frequency at a given position is the fraction of reads with a mutation and is displayed if >0.1 for each genomic position with sufficient sequencing depth. Within each window, a thick black line indicates the median value, and a blue bar indicates the interquartile range. Red lines indicate segmented values. The thin dotted lines indicate the copy number expectation values under the fitted model. **(H)** Visualization of CNV on chromosome 4 estimated by the software Sequenza (26) for the tissue (left panel) and the model (right panel). In each panel are reported two annotation layers. In the upper one dark-grey areas represent genes, while white segments are intergenic regions. The lower panel shows Giemsa staining, in particular Giemsa-positive bands are white, Giemsa-negative are light-grey and centromeric regions are pink. The dashed vertical lines show the position of KIT and TET2 genes.

To assess whether the established organoid culture captures the main genetic feature of the tissue, we performed whole-exome sequencing and in-depth genomic analysis. Of note, a different loco-regional lymph node metastasis was available for isolation of nucleic acids and therefore used for comparative analyses. The tissue and the culture displayed a relatively low and comparable total mutational burden (**Figure 2A**) (0.8 and 0.7 mutations per Mb for tissue and model, respectively), which is in line with available literature (14, 17, 30). Overall, a total of 30 somatic single-nucleotide variations (SNVs) were detected, and 17 (57%) were shared between the samples (**Figure 2B**, **Supplementary Table 2**). As expected, shared mutations were mostly clonal, while private mutations displayed low allele frequency of the variants suggesting ongoing clonal evolution at the two distinct sites (**Figure S2A**). Of note, the distribution of the variant allele frequencies (VAF) was centered around 50% for the organoid culture while was centered around 30% for the tissue due to contamination of other non-tumoral cells (**Figure S2B**). Next, we computed the mutational signatures from whole-exome sequencing data (see methods) (**Figure 2C**). Among all the COSMIC signatures, Age (S1) and BRCA (S2) were prevalent both in the organoid and the original tumor (**Figure 2C**). The comparison of detected variant type and SNV class between organoid and tissue also confirmed genetic conservation at single nucleotide level (**Figure S2C**). Among the SNVs detected, we observed a heterozygous nonsense mutation of *ARID2*, a heterozygous in frame deletion of *TOX3*, and a homozygous nonsense mutation of *AMBRA1* gene (**Figure 2D**). Of the genetic alterations identified, only the inactivation of *AMBRA1* has been previously shown to anticipate the pharmacological response to targeting agents, specifically the reduced activity of the CDK4/6 inhibitor Palbociclib (31). Therefore, we challenged the organoid culture for 72 hours with either Palbociclib or the mTORC1 inhibitor Everolimus, which is predicted to have some activity towards pancreatic but not small intestinal NETs (31). The cell viability of the PDOs was minimally affected by the treatment only at high doses of both drugs (**Figure 2E**). The highest dose of both Palbociclib and Everolimus was then added to the culture before measuring apoptotic cell death. We found no significant increase of apoptotic cell death in treated cells as compared to the untreated culture (**Figure S2D**), this suggesting that the two compounds had mainly a cytostatic effect at the tested dosage.

No mutation was detected in the well-established PanNETs drivers (*MEN1*, *ATRX* and *DAXX*), nor in the tumor suppressor genes *TP53* and *RB1* that are often inactivated in NEC. To further corroborate the lack of alterations in *TP53* and *RB1*, we performed immunohistochemical staining for the corresponding proteins in the organoid culture. Expression of RB1 could be observed in the nucleus of all cells composing the culture, thus excluding underlying macroscopic genetic alteration not detected at whole-exome sequencing (**Figure 2F**). p53 stained positive in few nuclei, which would suggest the activation due to replication stress in the culture (**Figure 2F**). Loss of nuclear staining for ATRX and DAXX has been proposed as a surrogate biomarker for the identification of PanNETs with inactivation of those genes (14, 32), which sometimes cannot be detected through sequencing. Furthermore, the loss of either ATRX and DAXX in PanNETs has been associated to the Alternative Lengthening of Telomeres (ALT) phenotype (33) and a peculiar pattern of whole-chromosomal losses (14). These events are not observed in intestinal neuroendocrine tumors and, accordingly, the organoid culture showed prominent nuclear expression of both ATRX and DAXX (**Figure S2E**). Copy-number variation analysis (**Figure 2G**) of the tissue and the derived organoid culture showed a comparable profile. Analysis highlighted a diploid asset and copy-number gains involving chromosome 4 for both samples (**Figure 2G**). The extent of the chromosome 4 gain was different between the two specimens, possibly reflecting on the different neoplastic cell content. Among genes located on the chromosome 4, *KIT* and *TET2* genes exhibited a copy-number gain of 5 and of 3 in the organoid culture and tissue specimen, respectively (**Figure 2H**).

### 3.4 Transcriptomic characterization of the siNET organoid culture

**Figure 3:**
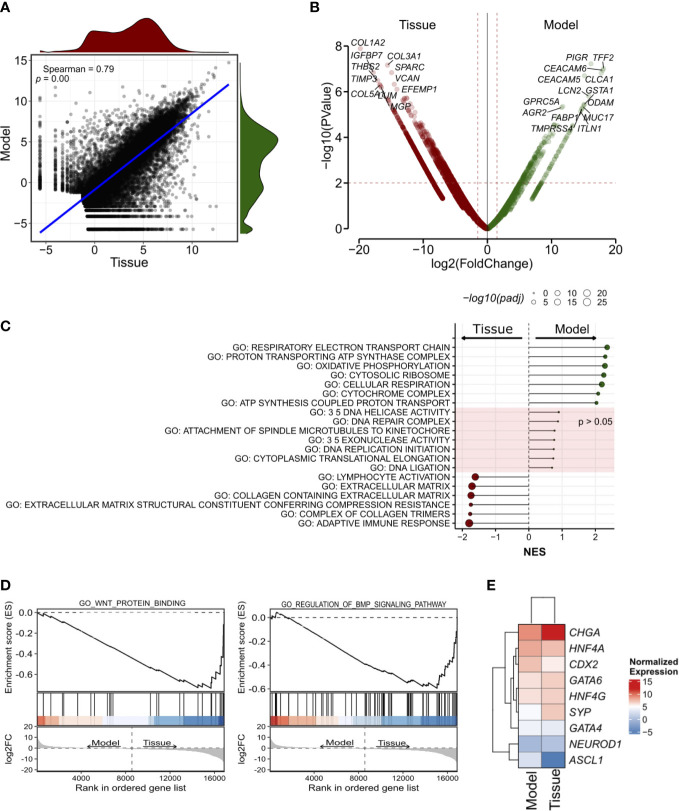
Transcriptomic characterization of the siNET organoid culture. **(A)** Scatter plot showing the correlation between the model and the tissue transcriptomes (Spearman’s correlation) across all 19,828 genes detected. The density plots show the distribution of gene expression in the siNET organoid (green) and the tissue (red). **(B)** Volcano plot of differences in gene expression between tissue and model. Indicated are some of the genes with |log_2_FoldChange| ≥ 2 and adjusted *p* < 0.01. See **Supplementary Table S3** for the complete list of differentially-expressed genes (DEGs). To account for the lack of biological and technical replicates in the identification of DEGs, we used a larger matrix of expression profiles of pancreatic ductal adenocarcinoma tissues and corresponding organoids. We used edgeR pipeline (27) to calculate a biological coefficient of variation (BCV) that ideally would represent an approximate biological variation for the siNET case. Squaring the BCV returned a dispersion value of 0.579 that was employed in the exactTest function to obtain p-values and fold-changes for each gene. **(C)** Selected GO pathways enriched in Model (green) or Tissue (red), or equally represented in both samples. GSEA was performed using gene sets from MsigDB library. See **Supplementary Table 4** for the complete list of tested pathways. **(D)** GSEA plot showing enrichment of pathways involved in WNT and BMP signaling in patient’s tissue. **(E)** Heatmap showing the expression of transcription factors of the gastrointestinal cell lineage and neuroendocrine tumor markers in Tissue and Model.

Next, we performed RNA-seq on the organoid culture and the tissue (**Figure 3**). First, we found a high correlation between the transcriptomic profiles of the two specimens (**Figure 3A**, see methods for details). *CHGA, HNF4A*, and *CDX2* were amongst the most expressed genes both in the model and in the tissue (**Figure S2F**) while ISL-1- a proposed marker of pancreatic neuroendocrine tumor (34, 35)- ranked amongst the low-expressed genes. IMP3 is reported to be an unfavorable marker in GI-NET, independent of the Ki67 status (36, 37). Therefore, we looked at the level of its transcript and found that it was amongst the less expressed genes in the model as well as in the tissue.

Then, we performed differential gene expression analysis to highlight genes and gene programs that differentiate the culture from the tissue (**Figure 3B**; **Supplementary Tables 3**, **4** ; see methods for details).

A Total of 1385 differentially expressed genes were identified using a p-value less than 0.01 and depicted in **Figure 3B** (see methods).

Genes encoding for Collagens (e.g., *COL1A2, COL3A1, COL5A1*), and other stromal associated proteins (e.g., *SPARC, VCAN*) were upregulated in the tissue and likely reflected the presence of stromal components, which are instead missing in the culture. Accordingly, gene set enrichment analysis on the differentially expressed genes identified enrichment of terms related to extracellular matrix and immune responses (**Figure 3C**; **Supplementary Table 4**). EMT was also linked to the tumor tissue specimen expression profile, together with *KRAS* signaling, myogenesis, apical junction components complex, coagulation and several immune-related pathways. Of note, we also observed enrichment of several signatures related to BMP (GO RESPONSE TO BMP, GO REGULATION OF BMP SIGNALING PATHWAY) and WNT (GO WNT PROTEIN BINDING, WP REGULATION OF WNT BCATENIN SIGNALING BY SMALL MOLECULE COMPOUNDS) signaling in the tumor tissue (**Figure 3D**).

Among the genes upregulated in the organoid culture, there were *PIGR*, *TFF2* and *CEACAM6* genes. Several genes encoding mucins (MUC2, MUC7, MUC5AC, MUC5B, MUC17), regenerating family member (REG1A, REG1B, REG3A, REG4), serine peptidase inhibitor kazal type (SPINK1, SPINK4, SPINK5) and glutathione-s-transferase A (GSTA1, GSTA2) family were similarly more expressed in this organoid model. Most of those genes are mainly contributed by the epithelium, and therefore enrichment of their transcripts in the organoid culture should be interpreted with caution and might simply reflect different neoplastic cell content. Furthermore, the differential expression of *ASCL1* and *CCND1* was observed in this sample. GSEA highlighted the enrichment of terms related to mitochondrial metabolism (**Figure 3C**) in the organoid culture, while there was no significant enrichment of terms related to cell cycle and DNA replication which corroborates the comparable fraction of proliferating cells as detected through Ki67 immunohistochemistry. Then, we focus on the analysis of transcription factors involved in specification and maintenance of gastrointestinal cells as well as of neuroendocrine tumor markers. We found similar expression of gastrointestinal lineage-specific transcription factors, such as *CDX2*, *GATA4*, *GATA6*, *HNF4A*, and *HNF4G*, in patient’s tissue and model transcriptome, with low expression of the neuronal transcription factors *ASCL1* and *NEUROD1* (**Figure 3E**). Of note, we confirmed the higher expression of *CHGA* and *SYP* in the tissue as observed in immunohistochemistry (**Figure 3E**). Several metabolic processes were associated to organoid expression profile (HALLMARK CHOLESTEROL HOMEOSTASIS; FATTY ACID METABOLISM; GLYCOLYSIS) as well as hypoxia and TP53 signatures (**Supplementary Table 4**).

## 4 Discussion

Long-term propagation of slow-proliferating (G1/G2) NETs organoids has not been reported so far (21, 38), and the available mouse and human NETs cell lines are poorly representative of the genetics of human well-differentiated tumors (20).

Here, we developed a tridimensional culture from a metastatic small intestinal NET using the organoid culture methodology (23, 24). Differently from previous experiences (20, 21, 38), this culture could be expanded for several months, resuscitated cryopreservation, and demonstrated to preserve major molecular features of the tissue. Furthermore, the culture permitted functional characterization, including drug screening.

To our knowledge, this is the first long-term organoid culture from a G2 NET. The PDO displayed a low proliferation index, which was comparable to that of the tissue, yet it could be propagated continuously over the course of 28 weeks without dramatic changes to the karyotype. In keeping with its grade and differently from carcinomas, the model demonstrated low tumorigenic potential *in vivo*. Overall, this suggest that the culture system did not increase the tumorigenicity of the model. After few weeks in culture, the PDOs could be used to test the activity of pharmacological compounds using the same procedure we and others have previously described (39–41).

Low number of total SNVs and the absence of alterations in known cancer-related genes reflect a typical genomic landscape of small intestinal neuroendocrine tumor (13, 17, 30, 42). Three deleterious alterations were identified in tissue and organoids to likely represent oncogenic events in this case: a heterozygous nonsense mutation of *ARID2*, a heterozygous in frame deletion of *TOX3*, and a homozygous nonsense mutation of *AMBRA1* gene.

*ARID2* encodes for a subunit of the PBAF chromatin remodeling complex and function as coregulators for nuclear receptors. ARID2 deficiency is reported to exert oncogenic effects in several malignancies (43–45).The *TOX3* gene encodes for a high mobility group box protein for which several functions have been reported including the regulation of calcium-mediated transcription (46). However, given the nature of the genetic alteration (in frame deletion), it is difficult to predict the functional consequences of the TOX3 alteration identified here.

*AMBRA1* gene encodes for a multifunctional scaffold protein that participate to the regulation of an array of biological processes, spanning from apoptosis to cell proliferation (47). Importantly, *AMBRA1* has been proposed to act as an haploinsufficient tumor suppressor that induces spontaneous tumors in animal models by regulating the stability of c-Myc (47) or Cyclin D1 (48). Previous works have demonstrated that the loss of AMBRA1 predicts poor response to the CDK4/6 inhibitors and, accordingly, the PDO showed poor sensitivity towards Palbociclib. Furthermore, the models also showed to be refractory to the treatment with the mTORC1 inhibitor Everolimus, which is commonly used for the treatment of PanNETs but not siNETs (49–51).

Inactivation of TP53 and RB1 appeared to be key requirements for the *ex vivo* propagation of neuroendocrine cells (21), and accordingly the genetic alterations of those genes are frequent in NEC. The integration of DNA-Sequencing, immunohistochemistry and functional assay showed that the siNET PDO is proficient for both TP53 and RB1. First, no evident genetic alteration could be detected through whole-exome sequencing for both *TP53* and *RB1.* Moreover, RB1 could be detected at protein level through immunohistochemistry and Palbociclib reduced proliferation of the siNET in cultures as expected for *RB1* wild-type cells. The immunohistochemical expression of TP53 could be detected in scattered cells composing individual organoids. Together with the lack of genetic evidence for the inactivation of the gene, that suggests induction of the protein upon replication stress in culture.

Alterations in chromosome asset is a hallmark of siNET (52). A substantial fraction of siNET shows loss of heterozygosity (LOH) of chromosome 18 or the acquisition of one or more copies of chromosome 4, 5, 7, 14 and 20 (42, 53, 54). In present study, we reported copy gain of chromosome 4 and in particular of *KIT* gene locus which was previously reported as altered in siNET (13, 42). In keeping with the tissue of origin, we did not detect alterations of ATRX and DAXX which are instead frequent in low-to-intermediate grade PanNETs.

RNAseq data comparison highlighted differences between the tissue and the organoid. Among differentially expressed genes, those related to extracellular matrix and immune response processes were enriched in the tissue. These genes and gene programs were not observed in the organoid culture, likely due to lack of microenvironmental components. Nevertheless, the expression of differentiation genes and pathways was mostly retained in the organoid culture, suggesting that the culture system did not lead to major dysregulation of the cell lineage.

In conclusion, we report for the first time of the long-term propagation of a human small intestinal NET as an organoid culture. We demonstrated that the PDO can be propagated continuously, retained key genetic and phenotypic features of the tissue, and could be readily subjected to functional characterization including pharmacological screens and *in vivo* transplantation to permit genotype-phenotype analysis.

## Data availability statement

The data presented in the study are deposited in the EGA European Genome-Phenome Archive (https://ega-archive.org/), accession numbers EGAS00001007093 (whole exome sequencing data) and EGAS00001007108 (RNA sequencing data).

## Ethics statement

The studies involving human participants were reviewed and approved by Ethics committee at University of Verona, Italy: approval number 1911 (Prot. n 61413, Prog 1911 on 19/09/2018) from the Integrated University Hospital Trust (AOUI) Ethics Committee (Comitato Etico Azienda Ospedaliera Universitaria Integrata). The patients/participants provided their written informed consent to participate in this study. The animal study was reviewed and approved by the Institutional Animal Care and Use Committee at University of Verona (Approval number: 655/2017-PR).

## Author contributions

SD’A, EF, and VC designed the research. SD’A, EF, CV, SA performed experiments. FP, PD, and MS analyzed data and generated displays. RTL, AP, CB, and LL collected samples and clinicopathological information. PC, BR, CL, and AS performed histopathological evaluation of human tissues and xenografts. SD’A, EF and VC wrote the manuscript. VC supervised the study. All authors approved the final version of the manuscript.

## Supplementary material

Supplementary Table 1 Neuroendocrine tumors enrolled in the study.

Supplementary Table 2 Whole-exome sequencing of the model and the tissue. **(A)** Non-synonymous somatic mutations detected in the model. **(B)** Non-synonymous somatic mutations detected in the tissue. The mutations shared between the model and the tissues are highlighted in yellow. Related to .

Supplementary Table 3 List of genes differentially expressed between the model and the tissue.

Supplementary Table 4 Gene set enrichment analysis based on genes differentially expressed in the comparison between model and tissue. Gene sets enriched in the tumor tissue and reported in are highlighted in light orange; gene sets enriched in model and reported in are highlighted in light green; gene sets that do not show enrichment in either tumor or model are highlighted in light blue.

Supplementary Figure 1 Characterization of siNET Organoid culture. **(A)** Representative H&E staining and immunohistochemistry for CDX2, CHGA, and SYP of an additional FFPE block of the pancreatic resected specimen. Scale bar, 300 µm. **(B)** Representative H&E staining and immunohistochemistry for CDX2, CHGA, and SYP of the organoid culture at passage 9, 15, and 24. Scale bar, 200 µm. **(C)** Representative photogram of mouse pancreas and spleen after necroscopy. Arrow indicates the site of organoids injection. **(D)** Representative H&E staining and immunohistochemistry for CK8-18, CHGA, SYP, CD56, and Ki67 of the tissue (top) and the paired organoid culture (bottom) from a neuroendocrine carcinoma (NEC). The quantification of Ki67+ cells (10 field of investigation, 20X) for the NEC PDO is provided in the scatter dot plot on the right. Scale bars, as indicated. **(E)** Representative H&E of two different pancreatic tissues from immunodeficient mice transplanted with the NEC PDOs. Scale bars, as indicated.

Supplementary Figure 2 Molecular characterization of the siNET Organoid culture. **(A)** Heatmap showing the variant allele frequency (VAF) of shared (common) and private variants from the comparative lesion sequencing analysis of the model and the tissue. **(B)** Kernel plot displaying the variant allele frequency (VAF) density of tissue (red) and model (blue). **(C)** Single nucleotide variants (SNVs) detected in the tissue (top) and the model (bottom) according to variant classification (left panels), mutation type (middle panels), and class (right panels). **(D)** Representative images of activated Caspase-3/7 (green) in siNET organoid culture treated with Everolimus (5µM), Palbociclib (5µM), and vehicle (DMSO). Scale bar: 200µm. **(E)** Representative immunohistochemistry for ATRX (top) and DAXX (bottom) showing nuclear expression of the proteins in the model. Scale bars, 100 µm. **(F)**. Expressed genes sorted according to the Z-scores for both tissue (left) and model (right). In red, neuroendocrine markers.
