# Supplementary figures and images for: Long-term organoid culture of a small intestinal neuroendocrine tumor
Source: Front Endocrinol (Lausanne). 2023 Apr 4;14:999792. doi: 10.3389/fendo.2023.999792 (PMC10112019; doi:10.3389/fendo.2023.999792)

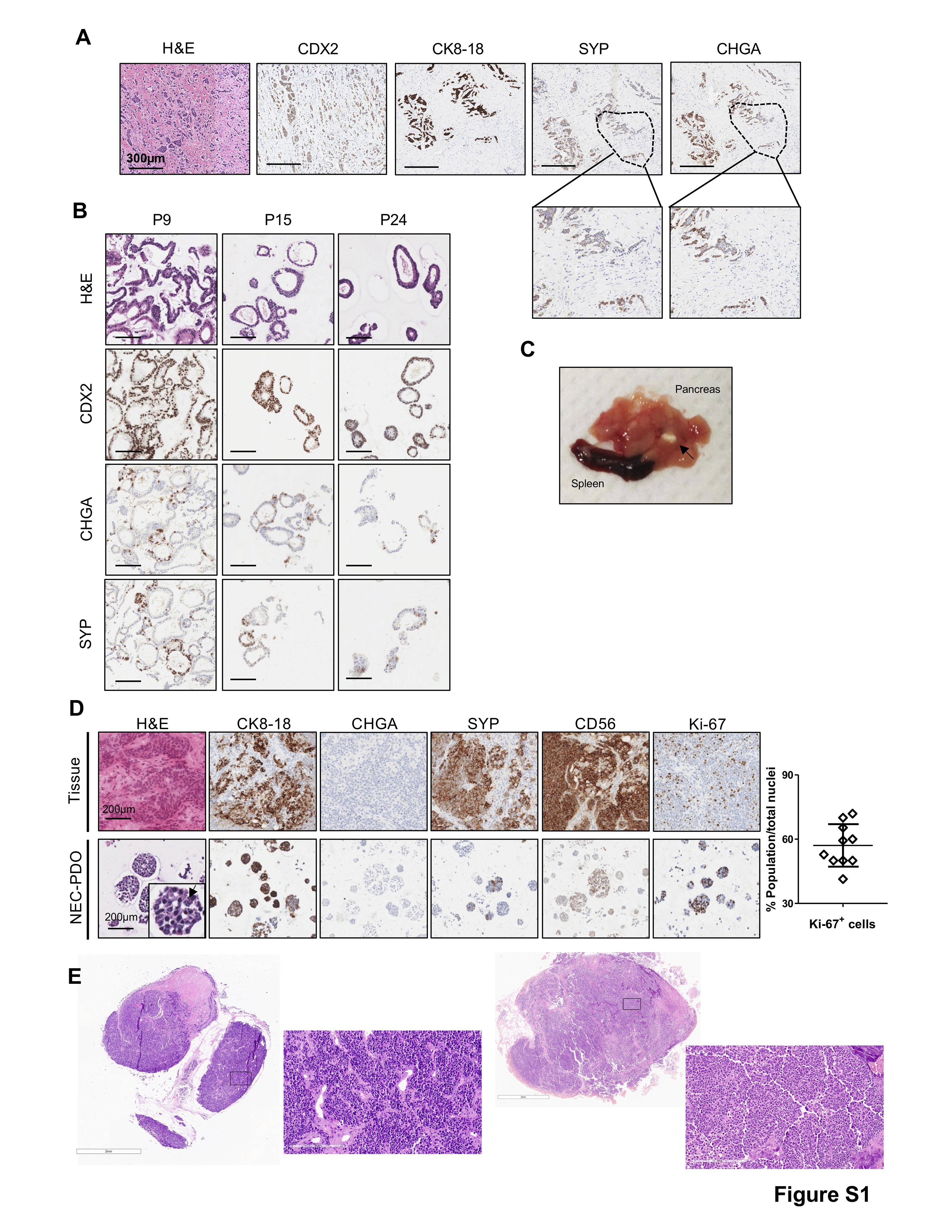

Supplement: Supplementary Figure 1 — Characterization of siNET Organoid culture. (A) Representative H&E staining and immunohistochemistry for CDX2, CHGA, and SYP of an additional FFPE block of the pancreatic resected specimen. Scale bar, 300 µm. (B) Representative H&E staining and immunohistochemistry for CDX2, CHGA, and SYP of the organoid culture at passage 9, 15, and 24. Scale bar, 200 µm. (C) Representative photogram of mouse pancreas and spleen after necroscopy. Arrow indicates the site of organoids injection. (D) Representative H&E staining and immunohistochemistry for CK8-18, CHGA, SYP, CD56, and Ki67 of the tissue (top) and the paired organoid culture (bottom) from a neuroendocrine carcinoma (NEC). The quantification of Ki67+ cells (10 field of investigation, 20X) for the NEC PDO is provided in the scatter dot plot on the right. Scale bars, as indicated. (E) Representative H&E of two different pancreatic tissues from immunodeficient mice transplanted with the NEC PDOs. Scale bars, as indicated. [file Image_1.jpeg]

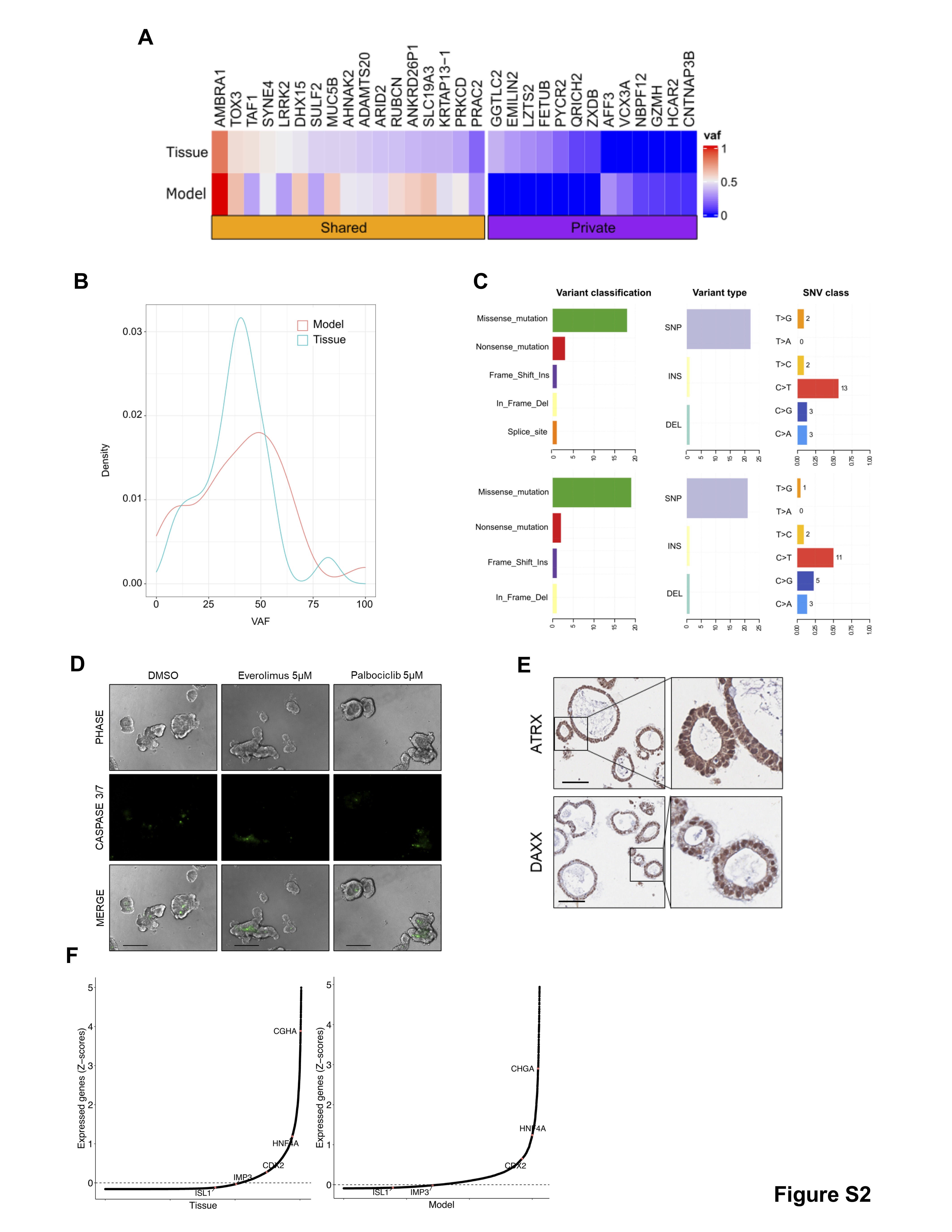

Supplement: Supplementary Figure 2 — Molecular characterization of the siNET Organoid culture. (A) Heatmap showing the variant allele frequency (VAF) of shared (common) and private variants from the comparative lesion sequencing analysis of the model and the tissue. (B) Kernel plot displaying the variant allele frequency (VAF) density of tissue (red) and model (blue). (C) Single nucleotide variants (SNVs) detected in the tissue (top) and the model (bottom) according to variant classification (left panels), mutation type (middle panels), and class (right panels). (D) Representative images of activated Caspase-3/7 (green) in siNET organoid culture treated with Everolimus (5µM), Palbociclib (5µM), and vehicle (DMSO). Scale bar: 200µm. (E) Representative immunohistochemistry for ATRX (top) and DAXX (bottom) showing nuclear expression of the proteins in the model. Scale bars, 100 µm. (F). Expressed genes sorted according to the Z-scores for both tissue (left) and model (right). In red, neuroendocrine markers. [file Image_2.jpeg]
